# KIAA0040 enhances glioma growth by controlling the JAK2/STAT3 signalling pathway

**DOI:** 10.1111/jcmm.18332

**Published:** 2024-04-25

**Authors:** Jie He, Kaming Xue, Fei Fan, Lin Li, Xinyu Rao, Wei Liu, Chuansheng Nie

**Affiliations:** ^1^ Department of Neurosurgery, Union Hospital, Tongji Medical College Huazhong University of Science and Technology Wuhan Hubei China; ^2^ Department of Traditional Chinese Medicine, Union Hospital, Tongji Medical College Huazhong University of Science and Technology Wuhan Hubei China; ^3^ Department of Information and Data Center, Union Hospital, Tongji Medical College Huazhong University of Science and Technology Wuhan Hubei China

**Keywords:** gliomas, JAK2/STAT3, KIAA0040, proliferation

## Abstract

The role of KIAA0040 role in glioma development is not yet understood despite its connection to nervous system diseases. In this study, KIAA0040 expression levels were evaluated using qRT‐PCR, WB and IHC, and functional assays were conducted to assess its impact on glioma progression, along with animal experiments. Moreover, WB was used to examine the impact of KIAA0040 on the JAK2/STAT3 signalling pathway. Our study found that KIAA0040 was increased in glioma and linked to tumour grade and poor clinical outcomes, serving as an independent prognostic factor. Functional assays showed that KIAA0040 enhances glioma growth, migration and invasion by activating the JAK2/STAT3 pathway. Of course, KIAA0040 enhances glioma growth by preventing tumour cell death and promoting cell cycle advancement. Our findings suggest that targeting KIAA0040 could be an effective treatment for glioma due to its role in promoting aggressive tumour behaviour and poor prognosis.

## 1 INTRODUCTION

Gliomas constitute the most prevalent and fatal brain malignancies.^1^ They are categorized into grades based on their level of malignancy according to the WHO classification.^2^ Grade 3 gliomas have around a 3‐year median survival time, while Grade 4 gliomas have 15 months.^3^ Gliomas symptoms depend on tumour location and can include headaches, vomiting, seizures and cranial nerve issues due to pressure. Despite improvements in multimodal therapy that includes surgery, radiation and chemotherapy, the outlook for glioma patients is still difficult.^4^, ^5^ Some genes have been found to be glioma biomarkers, which may help predict patient susceptibility and prognosis. Gliomas inhibit the immune system by producing high levels of immunosuppressive factors like PD‐L1 and IDO, which hinder immune responses against the tumor.^6^, ^7^

The newly found gene KIAA0040 is linked to a higher risk of alcohol dependence and contains several consistent SNP risk.^8^ Additionally, KIAA0040 expression is significantly associated with genes involved in neurotransmitter systems and metabolic pathways linked to AD.^9^ Previous studies have not connected KIAA0040 to glioma progression. Accordingly, more research is needed to investigate its potential role in the disease and its impact on progression. The JAK‐STAT3 pathway is important for cell signaling.^10^, ^11^, ^12^ The STAT proteins regulate essential biological processes, including differentiation, proliferation, apoptosis and inflammation.^13^, ^14^, ^15^ Phosphorylated STAT3 forms dimers and moves to the nucleus, activating genes that impact tumour cell behavior.^16^, ^17^ The JAK2/STAT3 pathway is important in many cancers, especially gliomas.^18^, ^19^, ^20^

Our study shows that overexpression of KIAA0040 is linked to glioma malignancy through the JAK2/STAT3 pathway. This research suggests a new treatment strategy for gliomas, but more studies are needed to confirm these results and investigate targeting the KIAA0040‐JAK/STAT3 axis for therapy.

## 2 MATERIALS AND METHODS

### 2.1 Bioinformatics database

The gene expression data of glioblastoma (GBM) was sourced from The Cancer Genome Atlas (TCGA) database. Following the acquisition, the data underwent processing via cBioPortal along with GraphPad Prism 7 Software (GraphPad Prism Software Inc., San Diego, CA, USA). For further elucidation, we refer readers to the accompanying relevant documentation.^21^

### 2.2 Cell lines and reagents

The widely utilized human GBM cell lines A172 and LN229 were procured from the American Type Culture Collection (Manassas, VA, USA). The cell line culture was performed in Dulbecco's Modified Eagle Medium (DMEM; Hyclone, Logan County, KY, USA) supplemented with 10% fetal bovine serum (FBS; Gibco, Grand Island, NE, USA) and 1% penicillin (100 U/L) and streptomycin (100 mg/L) (both from Gibco). Bosutinib (SKI‐606) as well as Anti‐KIAA0040 (HPA068812) antibodies, were acquired from Sigma‐Aldrich. Meanwhile, Anti‐JAK2 (ab108596), Anti‐P‐JAK (ab138005), anti‐STAT3 (ab109085), anti‐P‐STAT3 (ab267373), anti‐GAPDH (ab9485), and anti‐β‐actin (ab8227) antibodies were obtained from Abcam (Cambridge, MA, USA). For further details, we direct readers to the pertinent documentation for comprehensive solutions.^22^, ^23^, ^24^

### 2.3 Patients

Tumour samples were meticulously gathered from patients undergoing neurosurgical resection procedures for gliomas. Upon surgical removal, the tumour specimens were promptly handled and prepared for preservation. They were rapidly frozen using specialized techniques and stored at −80°C. This meticulous preservation process was implemented to ensure the molecular integrity of the samples, thereby facilitating comprehensive and accurate analyses in subsequent investigations. The collection of tumour samples from the patients followed ethical guidelines and regulations. Prior to the neurosurgical procedures, all patients provided informed consent for surgery and the anonymous scientific investigation of their pathological tissue. These consent forms ensured that the patients were aware that their tissue would be used for research purposes without disclosing their personal information. The guidelines and regulations for collecting and using human tissue samples in scientific research were granted approval from the Human Research Committee of Huazhong University of Science and Technology and the China Anti‐Cancer Association. These regulatory bodies oversee the ethical considerations and ensure that the rights and welfare of the patients are protected during the research process.

### 2.4 Plasmid constructs and transfection

Lentiviruses containing short hairpin RNAs (shRNAs) that target the KIAA0040 gene were obtained from Shandong ViGene (Shandong, China). These lentiviruses were used to infect cells to knock down the expression of KIAA0040. The shRNAs sequence used was 5′‐CATCAAATGGTCATTGGCTAT‐3′; 5′‐CGTGGACAATTCTTGGATACT‐3′. These shRNAs were designed to specifically bind to the mRNA of KIAA0040, leading to its degradation and reducing its expression in the infected cells. Cells infected with an empty vector or scrambled shRNA sequences were utilized for control experiments. These control groups help to assess the specificity and effectiveness of the gene knockdown by comparing the results to cells infected with the KIAA0040‐targeting shRNAs. Detailed solutions are available in the following relevant documents.^24^, ^25^, ^26^

### 2.5 Cell migration and invasion assays

Herein, we assessed cell migration and invasion through a transwell system (Corning, NY) per the protocols. To evaluate the cell invasive capability, the transwell filters were pre‐coated with Matrigel, a basement membrane matrix product (BD Biosciences, Jersey City, NJ, USA). These experiments were conducted with careful attention to detail, ensuring adherence to the specified procedures outlined below.

1. Pre‐coating of filters: The filters of the transwell system were coated with Matrigel before the experiment. Matrigel provides a three‐dimensional matrix miming the extracellular environment, allowing cells to invade and migrate.
2. Cell seeding: Approximately 1 × 10^4^ cells in serum‐free DMEM were added to the top chamber of the transwell system. The coated filter separates the top chamber from the bottom chamber, which acts as a barrier.
3. FBS‐containing medium: The bottom chamber was filled with DMEM that contained 20% FBS. The FBS is a chemoattractant, stimulating cell migration and invasion towards the lower chamber.
4. Incubation: The transwell system was incubated for 24 h, allowing the cells to migrate and invade through the Matrigel‐coated filter.
5. Removal of non‐invaded cells: Non‐migrating or non‐invading cells on the upper surface of the filter were delicately eliminated by a cotton swab.
6. Fixation and staining: Following fixation in 4% methanol for 15 min, the filter membrane was carefully prepared for staining with a 0.1% crystal violet solution, allowing for an incubation period of 30 min. The crystal violet solution effectively stains the cells that have traversed the Matrigel‐coated filter, marking their successful migration or invasion.
7. Cell counting: Meticulous cell counting was then performed, focusing on the migrated cells to the lower membrane surface. Under the scrutiny of a microscope, these cells were captured and enumerated with precision. This rigorous and systematic process provides a robust quantitative measure of the invasive or migratory capacity demonstrated by the cells under investigation.

### 2.6 Cell counting Kit‐8 (CCK‐8) assay

The cells were resuspended upon reaching the logarithmic growth phase, indicating a stage of active cell division. The cells went through seeding in 96‐well plates at a 5 × 10^3^ cells/well density. The culture medium used was incomplete, likely referring to the medium lacking specific additives or components required for specific experimental conditions. After seeding, the cells were allowed to adhere to the well surface for 24 h, allowing them to attach and establish a monolayer. After the designated incubation duration was completed, the culture medium was carefully eliminated, thereby treating the cells with CCK‐8 (10 μL) per the instructions. The CCK‐8 is a widely utilized reagent for assessing cell viability and proliferation in various experimental settings. This methodical approach ensures precise execution and accurate interpretation of the results obtained from the viability and proliferation assays. It measures the metabolic activity of cells, which is indicative of their viability. A microplate reader was used at a 450 nm wavelength to measure the absorbance. This specific wavelength is commonly used to detect the signal produced by CCK‐8, which correlates with cell viability. The baseline reading, which represents the background absorbance of the medium or reagents, was subtracted from the measured absorbance to obtain accurate results.

### 2.7 Colony formation assay

The colony formation ability of each cell line was assessed as follows.

1. Cell plating: 600 cells were plated in triplicate for each cell line into separate wells of 6‐well plates. Each well contained 3 mL of culture medium supplemented with 20% FBS to provide important nutrients and growth factors.
2. Incubation: The plated cells were incubated at 37°C with 5% CO_2_ for 2 weeks, with the culture medium remaining unchanged. The cells were allowed to grow and form colonies during this time.
3. Fixation: After the 2‐week incubation period, the cells were fixed with 4% formaldehyde. Formaldehyde preserves the cellular structure and prevents further growth or changes.
4. Staining: The cells were stained with crystal violet following fixation. Crystal violet is a commonly used dye that binds to cellular components, allowing for visualisation and quantification of the colonies formed.
5. Colony counting and photography: Colonies with a more than 2 mm diameter were counted manually under a microscope. The colonies were also photographed to document their appearance and size.
6. Replicate experiments: The entire procedure was performed in triplicate to ensure the reproducibility of the results.

### 2.8 Western blot (WB) analysis

Herein, we analysed protein expression levels as follows.

1. Cell lysis: The cells were lysed, and protein lysates were obtained. The lysates likely contained equal amounts of protein from each sample.
2. SDS‐PAGE: We loaded the protein lysates onto a 10% SDS‐PAGE gel and were subjected to electrophoresis. Electrophoresis separates proteins based on their molecular weight. The gel was run at 120 V for 2 h to ensure proper separation of the proteins.
3. Protein transfer: Following electrophoresis, the separated proteins were transferred to a polyvinylidene difluoride (PVDF) membrane to immobilize the proteins on the membrane, allowing for subsequent antibody detection.
4. Blocking: In the blocking step, the PVDF membrane underwent incubation with a blocking solution, typically consisting of 5% fat‐free milk dissolved in Tris‐buffered saline with Tween‐20. This crucial step effectively inhibits nonspecific binding of antibodies to the membrane surface, thereby enhancing the specificity and accuracy of subsequent immunodetection assays. By effectively blocking nonspecific binding sites, this meticulous procedure ensures the reliability and integrity of the experimental results obtained from the immunoblotting analysis.
5. Antibody incubation: Following the blocking step, the membrane underwent incubation with primary antibodies specifically targeting the protein(s) of interest. These primary antibodies are pivotal in recognising and binding to the target proteins present on the membrane surface. The membrane was thoroughly washed to eliminate any unbound antibodies after incubating with primary antibodies. This meticulous washing removes excess primary antibodies, thereby minimising background noise and enhancing the specificity of the immunodetection process.
6. Secondary antibody incubation: The membrane was incubated with secondary antibodies specific to the previously used primary antibodies. These secondary antibodies are coupled with enzymes or fluorophores for detection purposes.
7. Antibody detection: The membrane‐bound antibodies were detected through the Pierce Enhanced Chemiluminescence (ECL) detection system. The ECL generates a chemiluminescent signal when the enzyme‐conjugated secondary antibody reacts with specific substrates. This signal is typically captured using an x‐ray film or an imaging system.
8. Band analysis: The obtained x‐ray film or the captured image was analysed using Image J software, a widely used program for quantifying band densities in WB analysis. The software allows researchers to measure the intensity of the bands corresponding to the target proteins and compare their expression levels among different samples.

### 2.9 Immunohistochemical (IHC) and immunofluorescence staining (IFS) analyses

The process for IFS and IHC, as well as the scoring system used for evaluation, is described as follows:

IFS:

1. Tissue preparation: Tissue sections were fixed using 4% paraformaldehyde to preserve the tissue structure and proteins and permeabilized using 0.5% Triton X‐100, which allows the antibodies to penetrate the cells.
2. Washing: The tissue sections were rinsed three times using phosphate‐buffered saline to remove any residual fixative or permeabilization agent.
3. Blocking: The tissue sections were incubated in a blocking buffer that contained 5% bovine serum albumin to prevent nonspecific binding of antibodies.
4. Primary antibody incubation: Antibodies specific to the target proteins were suspended in the blocking buffer and incubated with the tissue sections overnight at a suitable temperature. These primary antibodies bind to their respective target proteins, enabling their detection.
5. Secondary antibody incubation: The tissue sections were incubated with secondary antibodies conjugated to Cy3 (a fluorescent dye) or another suitable fluorophore. These secondary antibodies recognize the primary antibodies and generate fluorescence signals.
6. Counterstaining and mounting: The tissue sections were exposed to counterstaining employing 4′,6‐diamidino‐2‐phenylindole (DAPI), which binds to DNA and helps visualize the cell nuclei. The sections were then mounted using an anti‐fade solution to preserve the fluorescence signals.
7. Imaging: Our study utilized a laser scanning confocal microscope (Olympus FV500 system) to visualize the fluorescence signals. This microscope captures high‐resolution images of the stained tissue sections.
8. Image processing: The acquired images were processed using Metamorph 2D deconvolution software, which helps enhance the image quality and visualize subcellular details.

IHC Scoring:

1. Tissue sections: The previously prepared formalin‐fixed and paraffin‐embedded tissue sections (4 μm thick) were utilized for IHC.
2. Primary antibody incubation: The tissue sections went through incubation with the primary antibody against the target protein, such as KIAA0040.
3. Secondary antibody incubation: The sections were incubated with a Cy3‐coupled secondary antibody specific to the primary antibody.
4. Scoring system: A semiquantitative scoring system known as the immunoreactive score (IRS) was employed to evaluate IHC staining results. The IRS is calculated by multiplying two key parameters: the staining intensity (SI) and the percentage of positive cells (PP). The SI was determined based on the intensity of observed staining, with scoring categories assigned as follows: 0 for negative, 1 for weak, 2 for moderate, 3 for strong, and 4 for very strong SI. Meanwhile, the PP was defined by the proportion of positively stained cells within the analysed tissue section, with scoring categories assigned as follows: 0 for less than 1%, 1 for 1%–10%, 2 for 11%–50%, 3 for 51%–80%, and 4 for more than 80% positive cells. These assigned SI and PP values were then multiplied to obtain the IRS, resulting in a final score ranging from 0 to 16.
5. Evaluation: Ten visual fields from distinct areas were meticulously evaluated for each sample to determine the IRS. This comprehensive approach ensures a representative assessment of protein expression levels across the entire tissue specimen. The scoring system considers the intensity and distribution of staining observed within the visual fields. Considering these two critical parameters, the scoring system comprehensively evaluates protein expression levels in the tissue sections under investigation.

### 2.10 Statistical analysis

The statistical analysis was conducted using the Empower Stats software program version 2.16.1 (San Diego, CA, USA). This study deployed a paired Student *t*‐test for comparative analysis to assess KIAA0040 mRNA levels across various human glioma grades. Clinical correlations were examined utilising two‐tailed Student's *t*‐tests. Survival analyses were conducted utilising log‐rank tests and Kaplan–Meier (K‐M) plots. Furthermore, we utilized the Cox regression model to conduct multivariate survival analyses.

## 3 RESULTS

### 3.1 KIAA0040 is highly expressed in tumour tissues and is inversely related to patient prognosis

**FIGURE 1:**
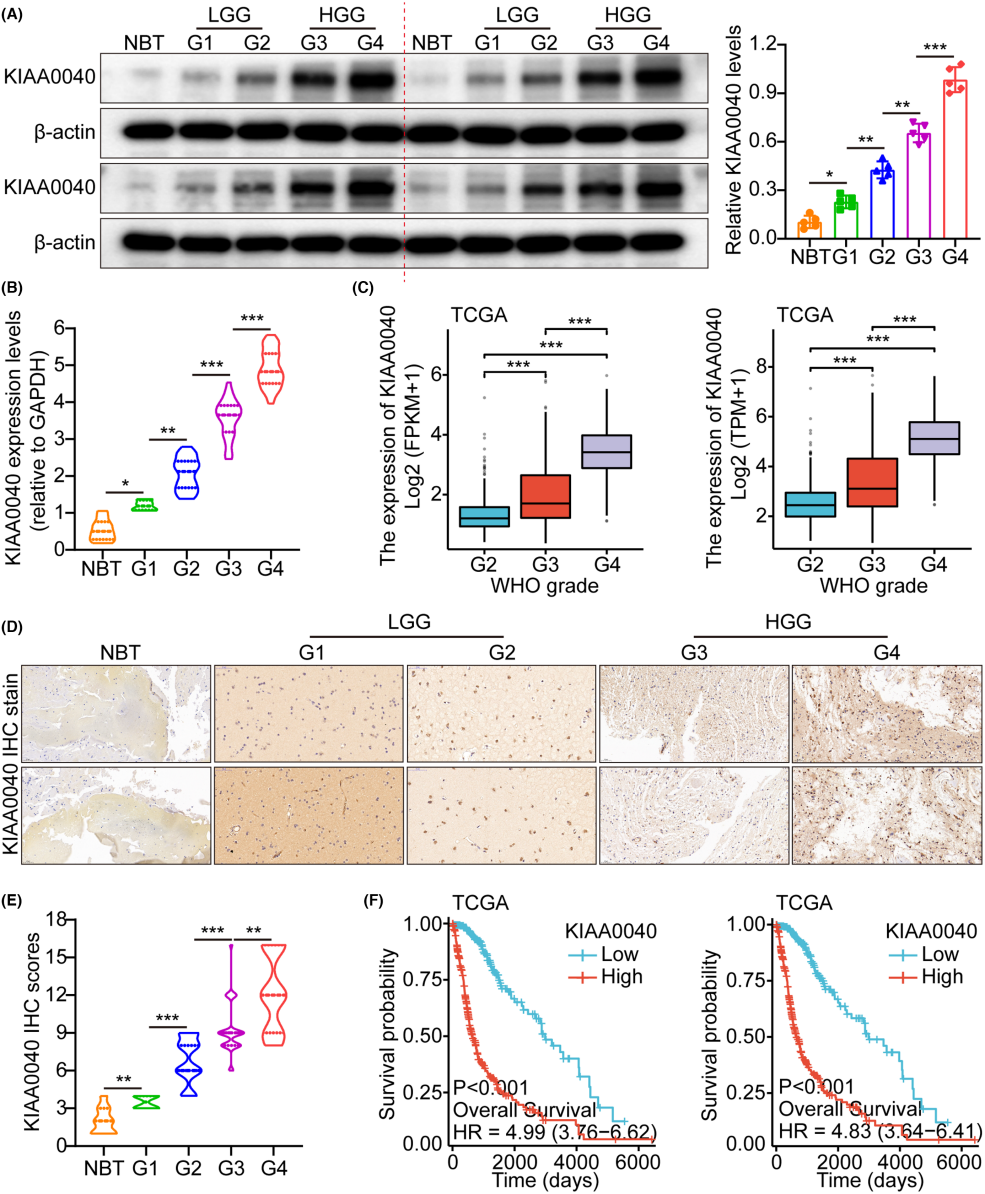
KIAA0040 is highly expressed in tumour tissues and is inversely related to patient prognosis. (A) WB was used to detect the expression levels of KIAA0040 in normal brain tissue (NBT) and gliomas of different grades (LGG (G1 + G2): low‐grade gliomas; HGG (G3 + G4): high‐grade gliomas). (B) qRT‐PCR was used to detect KIAA0040 mRNA levels in normal brain tissue and gliomas of different grades. (C) TCGA database results showed that KIAA0040 increases with increasing tumour grade. (D‐E) IHC staining and scoring were used to detect the protein levels of KIAA0040 in normal brain tissue and gliomas of different grades. (F) Log‐rank test of OS was conducted. The results of the TCGA database showed that the expression level was inversely proportional to the prognosis of patients. Data are presented as Mean ± s.d from three independent experiments. *p < 0.05, **p < 0.01 and ***p < 0.001.

The study utilized the glioma whole gene expression map database of the TCGA, which provided the genome sequence information of 5 samples of normal brain tissue (NBT) and 701 glioma tissues. The analysis results demonstrated that KIAA0040 was overexpressed in glioma tissues compared to NBTs. Herein, we only use two standardized methods, FPKM and TPM, for quantitative grouping, and adopt different statistical assumptions. Currently, TPM is more recommended. These two pictures actually express the same meaning (Figure S1A). This finding suggests that KIAA0040 is upregulated in gliomas, indicating its potential involvement in gliomas development. The RT‐PCR analysis was performed to investigate further the expression of KIAA0040 in different grades of human gliomas. The results showed that KIAA0040 expression varied across different grades of gliomas (Figure S1B). Additionally, WB analysis was conducted on 25 glioma tissues, including NBTs and grades I/II/III/IV gliomas. The WB results indicated that glioma tissues had higher KIAA0040 expression than NBTs. Moreover, there was a correlation between higher glioma grades and overexpressed KIAA0040 at the RNA and protein levels (Figure 1A–C). Herein, we only use two standardized methods, FPKM and TPM, for quantitative grouping, and adopt different statistical assumptions. Currently, TPM is more recommended. These two pictures actually express the same meaning (Figure 1C). The IHC analysis results showed that the immune SI of KIAA0040 was significantly different among different glioma grades (Figure 1D). Quantification analyses further supported the observation of increased KIAA0040 protein expression (Figure 1E). Furthermore, K‐M analysis demonstrated that higher KIAA0040 expression was related to shorter survival time (Figure 1F and Figure S1C). Herein, we only use two standardized methods, FPKM and TPM, for quantitative grouping, and adopt different statistical assumptions. Currently, TPM is more recommended. These two pictures actually express the same meaning (Figure 1F). This implies the potential role of KIAA0040 as a prognostic marker in gliomas. The study also explored the association of KIAA0040 expression with age, grade, and gender. The results found that higher KIAA0040 expression was correlated with older age and higher glioma grade (Figure S1D). However, no evidence supported an association between KIAA0040 expression and gender (Figure S1E–G).

### 3.2 Overexpression of KIAA0040 promotes glioma cell proliferation and invasion

**FIGURE 2:**
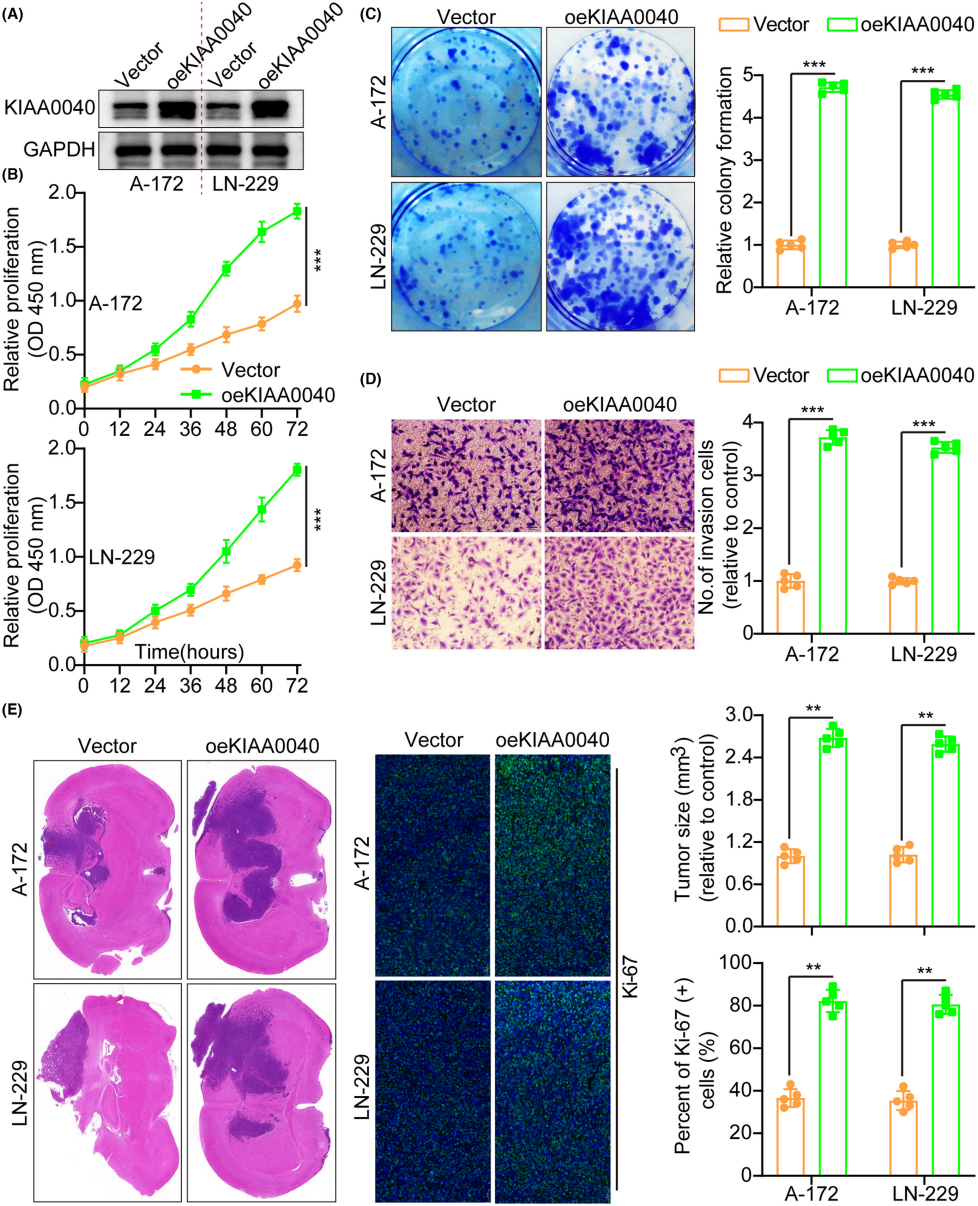
Overexpression of KIAA0040 promotes glioma cell proliferation and invasion. (A) Using WB to detect the overexpression efficiency of KIAA0040. (B) Cell growth curves measured by CCK‐8 between Vector and oeKIAA0040. (C) KIAA0040 overexpression facilitated colony formation and histogram quantification (panels). (D) Transwell migration and invasion assays show that overexpression of KIAA0040 facilitates cell migration and invasion. The numbers of migrants and invading cells. Bars: 50 μm. (E) Representative histogram of tumour size and Ki‐67 staining between Vector and oeKIAA0040. Data are presented as Mean ± s.d from three independent experiments. **p < 0.01 and ***p < 0.001.

Our study examined KIAA0040 expression in several cell lines to investigate its role in tumour cells (Figure S1H). Subsequently, A172 and LN229 cells were transfected with lentiviruses containing oeKIAA0040 to overexpress KIAA0040, confirming KIAA0040 expression using WB (Figure 2A). Moreover, we conducted various assays to assess the impact of KIAA0040 on the potential of glioma cells to proliferate and invade. The CCK8 assay, which measures cell viability and proliferation, depicted that KIAA0040 overexpression significantly enhanced glioma cell proliferation (Figure 2B). Similarly, the colony formation assay, which evaluates the ability of cells to form colonies, demonstrated that KIAA0040 overexpression enhanced the colony formation capacity of glioma cells (Figure 2C). Furthermore, the results of the transwell assay revealed that KIAA0040 overexpression significantly raised glioma cell invasion and migration (Figure 2D and Figure S2A). This indicates that KIAA0040 may promote the glioma cell invasive behaviour, a characteristic feature of malignant tumours. For further investigation of the influence of KIAA0040, annexin V staining and propidium iodide (PI) staining were performed (Figure S2B,C). Annexin V staining is commonly used to detect apoptotic cells, while PI staining is used to assess the cell cycle status. The results demonstrated that KIAA0040 overexpression led to a decrease in cell apoptosis, indicating a potential anti‐apoptotic effect. Of course, oeKIAA0040 also promotes the cell cycle progression (Figure S2D). Although the role of KIAA0040 in glioma cell lines was established, its role in vivo remained unclear. Therefore, cell lines with stable KIAA0040 overexpression and vector control were developed and directly injected into nude mice in situ. After 2 weeks, the mice were euthanized, and their brains were excised, weighed, and subjected to staining (Figure 2E). The IFS of Ki‐67, a marker for cellular proliferation, was performed on the brain tumours. The results demonstrated that KIAA0040 overexpression promoted Ki‐67 expression in comparison to the control group. The Ki‐67 is commonly used as an indicator of cell proliferation, and increased expression suggests enhanced tumour growth and progression. These findings strongly support the notion that KIAA0040 overexpression can reduce cell apoptosis and promote tumour progression in vitro and in vivo.

### 3.3 Knockdown of KIAA0040 suppresses glioma cell proliferation and invasion

**FIGURE 3:**
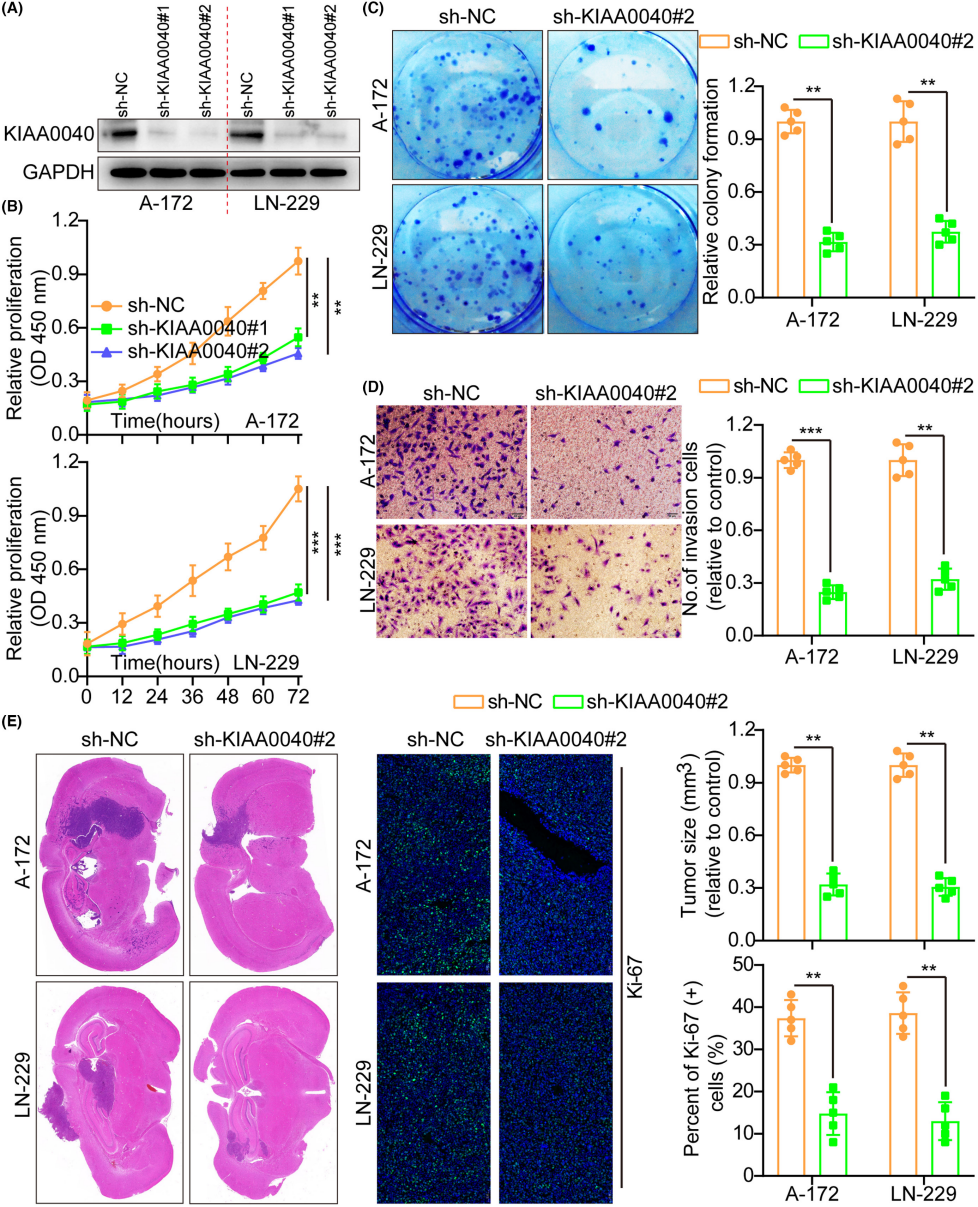
Knockdown of KIAA0040 suppresses glioma cell proliferation and invasion. (A) Using WB to detect the knockdown efficiency of KIAA0040. (B) Cell growth curves measured by CCK‐8 assay between sh‐NC and sh‐KIAA0040#2. (C) KIAA0040 knockdown inhibited colony formation and histogram quantification (panels). (D) Transwell migration and invasion assays showed that the knockdown of KIAA0040 inhibited cell migration and invasion. The numbers of migrants and invading cells. Bars: 50 μm. (E) Representative histogram of tumour size and Ki‐67 staining between sh‐NC and sh‐KIAA0040#2. Data are presented as Mean ± s.d from three independent experiments. **p< 0.01 and ***p < 0.001.

Here, we examined the effects of KIAA0040 knockdown in glioma cells utilising shRNAs targeting KIAA0040 in A172 and LN229 cells. The transfection efficiency of the shRNAs was confirmed by WB (Figure 3A). Functional assays, including the CCK8, colony formation, and transwell assays, were conducted to assess the impact of KIAA0040 knockdown on the proliferation and invasion potential of glioma cells. The results manifested that silencing KIAA0040 significantly impeded glioma cell proliferation and invasion (Figure 3B–D). This indicates that KIAA0040 is crucial in promoting glioma cell proliferation and invasion. Besides, the flow cytometric study results demonstrated that KIAA0040 knockdown promotes glioma cell apoptosis and halts the cell cycle progression (Figures S2C and S3A). Furthermore, we injected stable cell lines with KIAA0040 knockdown into nude mice in situ. After 2 weeks, the mice were euthanized, and their brains were excised, weighed, and subjected to staining (Figure 3E). The IFS of Ki‐67 was performed on the brain tumours, elucidating that KIAA0040 knockdown suppressed Ki‐67 expression in comparison to the control group. This suggests that inhibiting KIAA0040 reduced cellular proliferation in vivo.

### 3.4 Overexpression of KIAA0040 promotes glioma cell proliferation and invasion through JAK2/STAT3 pathway activation

**FIGURE 4:**
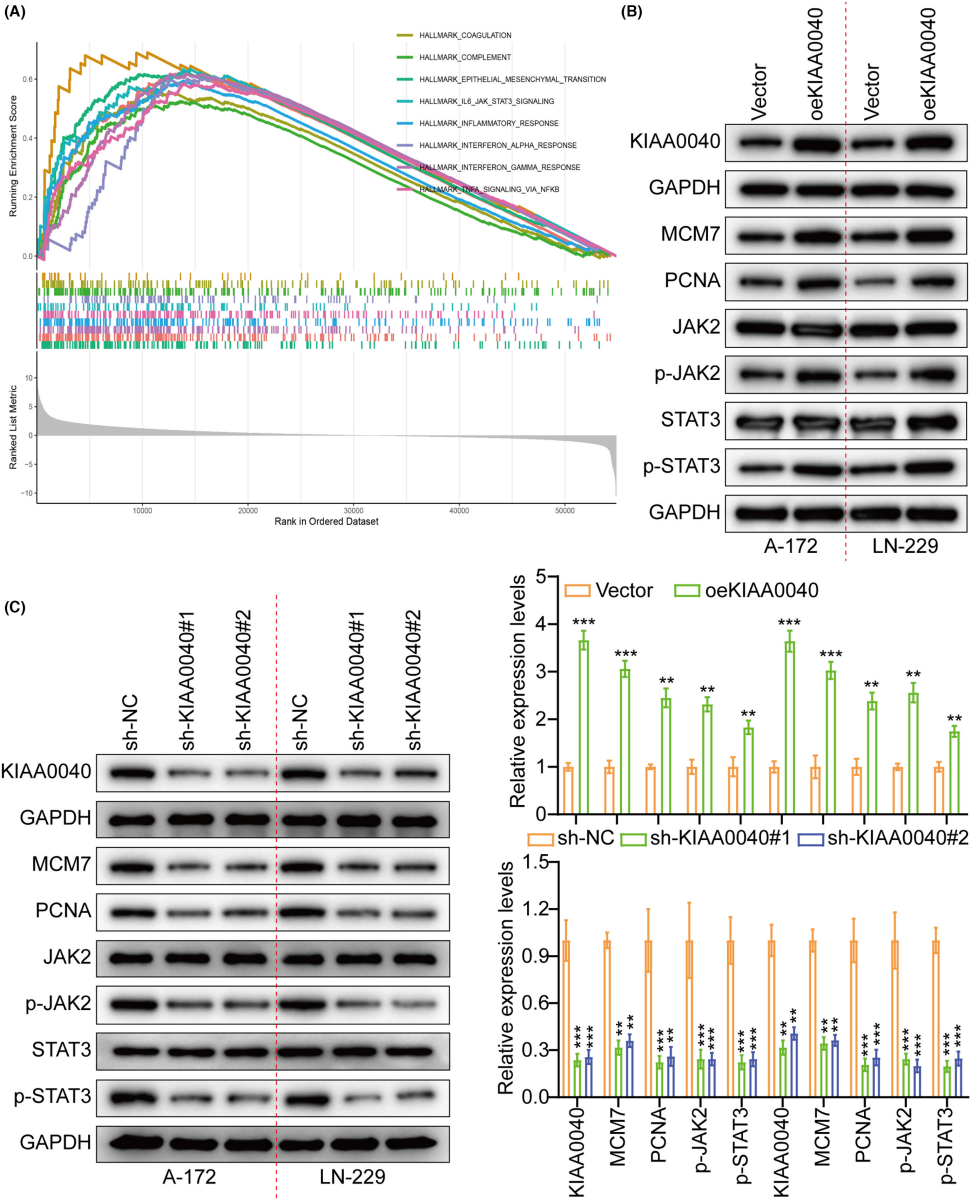
(A) GSEA results show that KIAA0040 is positively related to JAK2/STAT3. (B‐C) Expression of KIAA0040, MCM7, PCNA, JAK2, p‐JAK2, STAT3, and p‐STAT3 were detected by WB between sh‐NC, sh‐KIAA0040#1, sh‐KIAA0040#2, Vector, and oeKIAA0040. The downside and left represents the histogram. **p <0.01 and ***p <0.001.

**FIGURE 5:**
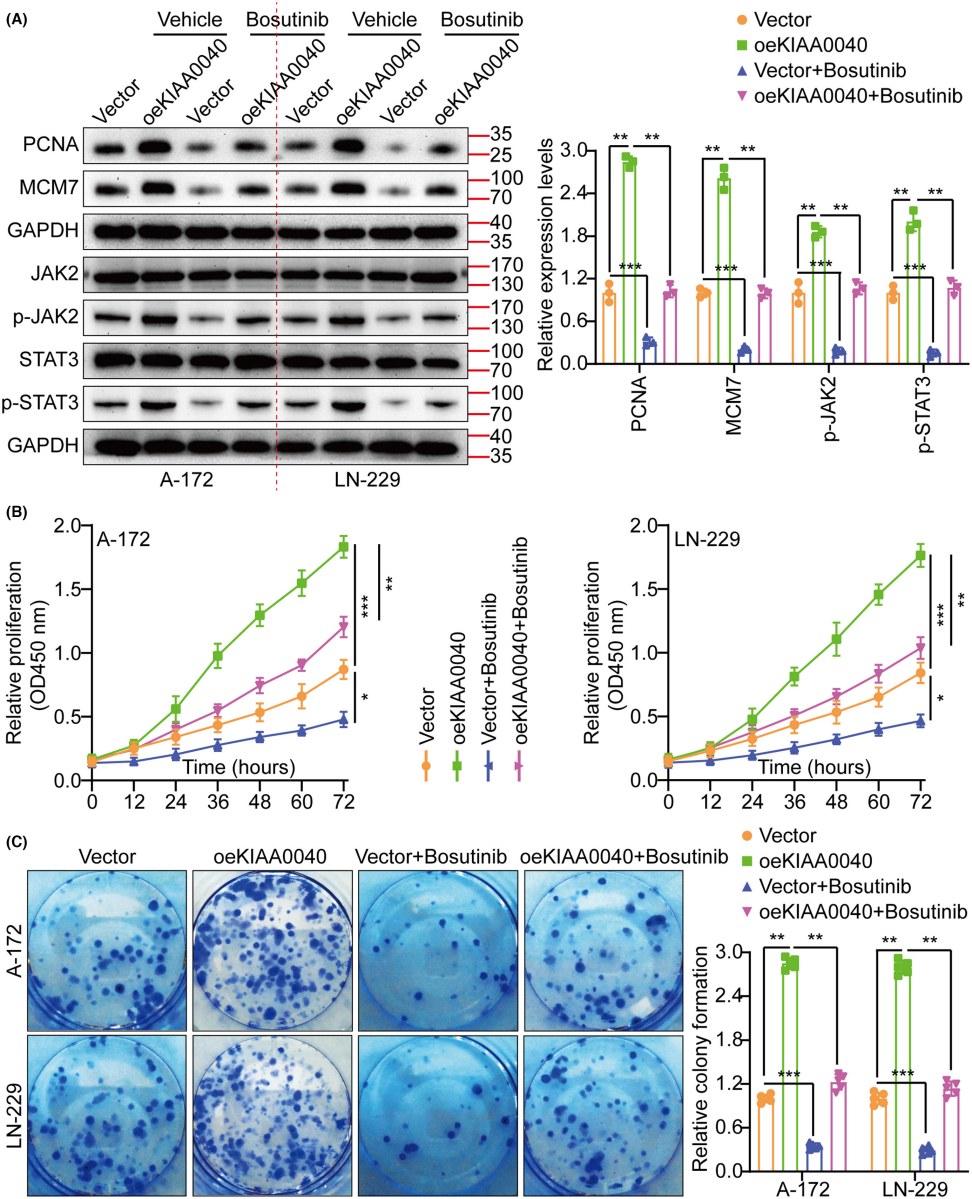
(A) Expression of MCM7, PCNA, JAK2, p‐JAK2, STAT3, and p‐STAT3 were detected by WB between Vector, oeKIAA0040, Vector + Bosutinib, and oeKIAA0040 + Bosutinib. (B) Cell growth curves measured by CCK‐8 assay in different treatment groups. (C) Cell growth was measured by colony formation assay in different treatment groups and histogram quantification (panels). Data are presented as Mean ± s.d from three independent experiments. *p < 0.05, **p< 0.01 and ***p < 0.001.

**FIGURE 6:**
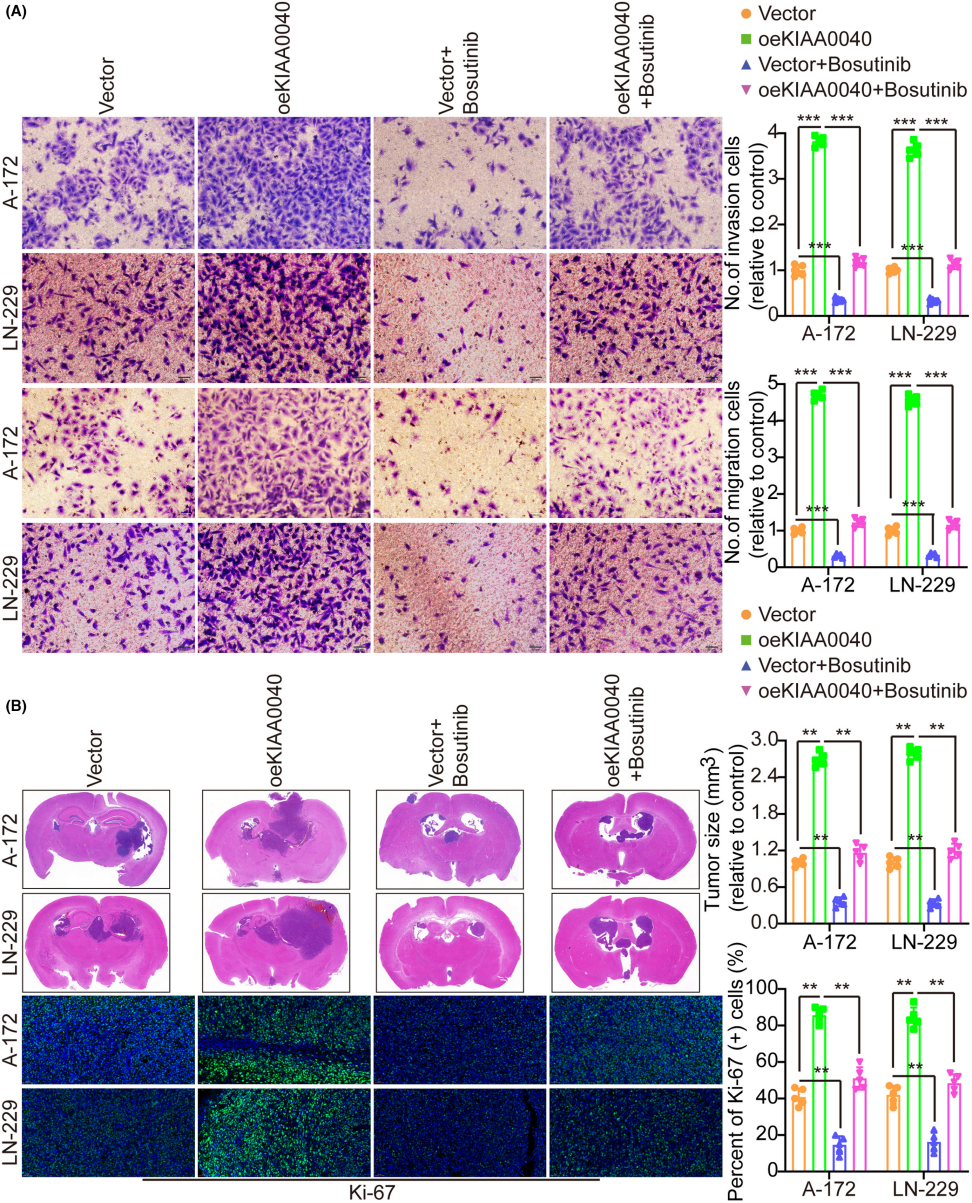
Overexpression of KIAA0040 promotes glioma cell migration and invasion through JAK2/STAT3 pathway activation. (A) KIAA0040 overexpression could promote tumour cell migration and invasion, and the effect could be attenuated by JAK2‐STAT3 inhibitors Bosutinib. The left side represents the histogram. (B) KIAA0040 overexpression could promote tumour cell growth, and the effect could be attenuated by JAK2‐STAT3 inhibitors Bosutinib. Data are presented as Mean ± s.d from three independent experiments. **p < 0.01 and ***p < 0.001.

A gene pathway enrichment analysis was conducted to elucidate the molecular mechanism by which KIAA0040 promotes glioma cell proliferation and invasion. The results manifested that KIAA0040 significantly influences the JAK2/STAT3 pathway (Figure 4A). Stable cell lines with KIAA0040 knockdown and overexpression were established to investigate this further, besides assessing the JAK2/STAT3 pathway activation (Figure 4B,C). The findings represented that KIAA0040 overexpression activated the JAK2/STAT3 pathway, while KIAA0040 knockdown suppressed this activation. The KIAA0040‐overexpressing cells were co‐cultured with Bosutinib, a JAK2/STAT3 pathway chemical inhibitor, to provide further evidence. Our findings showed that KIAA0040 overexpression significantly activated the JAK2/STAT3 pathway. However, the JAK2/STAT3 pathway activation was reversed when co‐cultured with Bosutinib. This suggests that KIAA0040 overexpression activates the JAK2/STAT3 pathway, and Bosutinib can attenuate this activation. Moreover, the promotion of cell proliferation by KIAA0040 was reduced in the presence of Bosutinib (Figure 5A–C). Bosutinib treatment significantly decreased the migration and invasion potential of A172 and LN229 cells, although it did not completely abolish the effect of KIAA0040 (Figure 6A). Flow cytometric analysis results revealed that Bosutinib reduced the effect of KIAA0040 on cell apoptosis and restored the cell cycle to control (Figures S3B,C and S4A). Furthermore, stable cell lines, either containing Bosutinib or not, were injected into nude mice in situ, and the brain tumours were examined after 2 weeks of growth (Figure 6B). The IFS of Ki‐67 in the brain tumours depicted that KIAA0040 overexpression resulted in larger tumour size and increased Ki‐67 expression in comparison to the control group. Nonetheless, under the effect of Bosutinib, both the tumour size and the expression of Ki‐67 were reduced to levels similar to control.

### 3.5 Bioinformatics analysis of KIAA0040

Next, we used the TCGA database to conduct a related pan‐cancer analysis of KIAA0040, the results show that KIAA0040 is highly expressed in most tumour tissues (Figure S4B). In addition, we also verified the expression abundance of KIAA0040 in 37 normal tissues in the GTEx database; the expression abundance of KIAA0040 in 18 immune cells and PBMC in the HPA database; and the expression abundance of KIAA0040 in solid tumour tissues and paracancerous tissues in the TCGA database. Expression differences and the prognostic value (OS) of KIAA0040 in solid tumours in the TCGA database (Figure S5A). Of course, we also verified the expression abundance of KIAA0040 in various cell subpopulations in low‐grade and high‐grade glioma single cell cohorts (Figures S5B and S6A). In addition, we also verified the correlation between KIAA0040 and immune cell infiltration abundance in low‐grade and high‐grade gliomas (Figure S6B). Of course, we also add more data on the expression of KIAA0040 protein in NBTs (Figure S6C). Indeed, the JAK family involves JAK1 and JAK2. We also tested the expression levels of JAK1 and p‐JAK1 after overexpressing KIAA0040, and found that compared with the control, there was no significant difference in the expression levels of JAK1 and p‐JAK1 after overexpressing KIAA0040 (Figure S6D). Finally, we used the TCGA database to verify the correlation analysis between KIAA0040 and all protein‐coding genes, and ranked them in descending order based on the correlation coefficient to perform GSEA enrichment analysis of 50 Hallmark pathways (Figure S7A).

## 4 DISCUSSION

Malignant gliomas present a substantial challenge due to their high aggressiveness and invasiveness as brain tumours. Despite the substantial advances in comprehending the tumour molecular mechanisms, our knowledge of the invasion and metastasis processes in gliomas remains limited. To improve patient outcomes, identifying and targeting potential therapeutic targets are crucial. Researchers are actively developing novel strategies to combat gliomas, and combination therapies are emerging as a promising approach to enhance the efficacy of immunotherapy. By combining different treatment modalities, such as chemotherapy, radiotherapy, targeted therapies, and immunotherapy, researchers aim to target multiple pathways and overcome treatment resistance synergistically. This multifaceted approach holds great promise for improving patient outcomes.^27^ In this context, discovering new therapeutic targets is of utmost importance. Identifying as many potential targets as possible enables researchers to explore different avenues for treatment. Researchers can uncover novel targets that may play critical roles in these processes by investigating the underlying molecular mechanisms of glioma invasion and metastasis. Such targets could be explored to develop innovative therapies to inhibit tumour invasion and metastasis.

The development and progression of gliomas involve a complex interplay between tumour suppressor genes and oncogenes. This study investigated the oncogenic role of KIAA0040 in glioma progression. Our findings revealed that KIAA0040 overexpression was significantly related to larger tumour size and poorer survival outcomes. By demonstrating the influence of KIAA0040 overexpression on glioma cell proliferation and invasion, we offer evidence for the strong tumorigenicity of KIAA0040 in gliomas. The results proposed that KIAA0040 is crucial in driving glioma malignant progression. The association between KIAA0040 overexpression and the cascade of glioma progression indicates its potential as a key contributor to glioma development and aggressive behaviour. The molecular mechanisms underlying the role of KIAA0040 in gliomas still require further investigation. Accordingly, we concluded that the oncogenic properties of KIAA0040 are at least partially attributed to its ability to enhance the JAK2/STAT3 pathway activity. The JAK2/STAT3 pathway possesses oncogenic functions, including promoting cell growth, enhancing migratory ability, and inducing drug resistance in cancer cells. Inhibiting the STAT3 signalling pathway has been elucidated to impede cancer progression and enhance the efficacy of chemotherapy‐induced cell death. Consistently, we demonstrate that KIAA0040 overexpression promotes glioma cell migration and invasion, which is possibly mediated via the JAK2/STAT3 pathway activation. These findings suggest that targeting KIAA0040 or the downstream JAK2/STAT3 pathway could be a potential glioma therapeutic strategy.

KIAA0040 is a gene located on chromosome 16 in humans. It encodes a protein that is involved in various cellular processes, although its specific functions are still being studied and understood. Mutations or dysregulation of KIAA0040 have been implicated in certain diseases, but further research is needed to fully elucidate its role in cellular biology and disease mechanisms.

Altogether, our results indicate that KIAA0040 promotes glioma cell migration and invasion through the JAK2/STAT3 pathway activation. This highlights the potential of KIAA0040 as a therapeutic glioma target and emphasizes the need for further research to elucidate its precise molecular mechanisms and explore its therapeutic implications.

## AUTHOR CONTRIBUTIONS

**Jie He:** Conceptualization (equal); data curation (equal); formal analysis (lead); funding acquisition (equal); investigation (equal); methodology (equal); resources (equal); software (equal); supervision (equal); validation (equal); visualization (equal); writing – original draft (lead); writing – review and editing (equal). **Kaming Xue:** Data curation (equal); formal analysis (equal); funding acquisition (equal); resources (equal); supervision (equal); validation (equal); visualization (equal); writing – original draft (equal); writing – review and editing (equal). **Fei Fan:** Conceptualization (equal); data curation (equal); formal analysis (equal); resources (equal); software (equal); supervision (equal); validation (equal); visualization (equal); writing – original draft (equal); writing – review and editing (equal). **Lin Li:** Conceptualization (equal); data curation (supporting); formal analysis (supporting); funding acquisition (supporting); investigation (supporting); methodology (supporting); validation (supporting). **Xinyu Rao:** Conceptualization (supporting); data curation (supporting); formal analysis (equal); investigation (equal); methodology (equal); software (equal). **Wei Liu:** Conceptualization (equal); data curation (equal); formal analysis (equal); funding acquisition (equal); investigation (equal); methodology (equal); project administration (equal); resources (equal); software (lead); supervision (equal); validation (equal); visualization (equal); writing – original draft (equal); writing – review and editing (equal). **Chuansheng Nie:** Conceptualization (equal); data curation (equal); formal analysis (equal); funding acquisition (equal); investigation (equal); methodology (equal); project administration (lead); resources (equal); software (equal); supervision (equal); validation (equal); visualization (equal); writing – original draft (equal); writing – review and editing (equal).

## CONFLICT OF INTEREST STATEMENT

The authors declare that they have no competing interests.

## Supporting information


Figure S1.

## Data Availability

The datasets used during the present study are available from the corresponding author upon reasonable request.
