# Supplementary material for: KIAA0040 enhances glioma growth by controlling the JAK2/STAT3 signalling pathway
Source: J Cell Mol Med. 2024 Apr 25;28(8):e18332. doi: 10.1111/jcmm.18332 (PMC11044867; doi:10.1111/jcmm.18332)

**KIAA0040 enhances gliomas growth by controlling the JAK/STAT3 signaling pathway**

Jie He^1*^, Kaming Xue^2*^, Fei Fan^1*^, Lin Li^1^, Xinyu Rao^1^, Wei Liu^3#^, Chuansheng Nie^1#^

**Figure Legends**

Supplementary Figure 1: Bioinformatics analysis of KIAA0040.

**A-C.** TCGA database analysis results show that KIAA0040 is highly expressed in tumor tissues, increases with tumor grade, and is inversely proportional to patient prognosis.

**D.** The heat map of TCGA database analysis results shows that the expression of KIAA0040 is positively correlated with patient age and tumor grade.

**E.** TCGA database analysis results show that KIAA0040 predicts patient survival time consistent with observed survival time.

**F.** The heat map of TCGA database analysis results shows that the expression of KIAA0040 is positively correlated with patient age.

**G.** TCGA database analysis results show the sensitivity of KIAA0040 in predicting patient survival time.

**H.** The relative expression levels of KIAA0040 in NHA and tumor cell lines.

Statistical significance was tested using one-way ANOVA (Dunnett’s tests) for multiple comparison and two-tailed t-tests. **P* < 0.05, ****P* < 0.001 and *****P* < 0.0001.


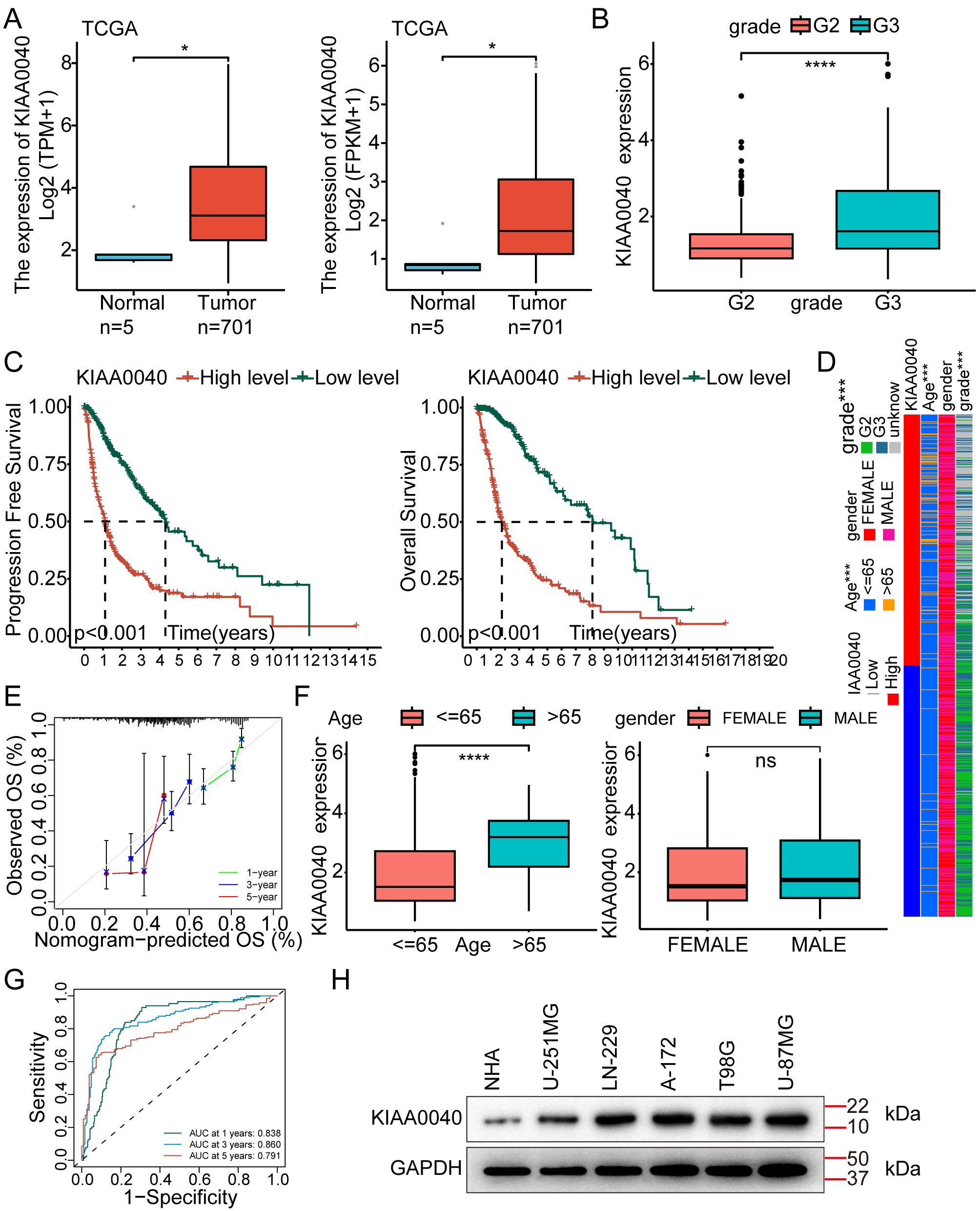


Supplementary Figure 2: KIAA0040 promotes tumor proliferation by inhibiting apoptosis and promoting cell cycle progression.

**A.** Transwell migration assays showed that overexpression of KIAA0040 facilitates glioma cell migration and the knockdown of KIAA0040 inhibited cell migration. The numbers of migration cells. Bars: 50 µm.

**B-D.** Flow cytometry results showed that overexpression of KIAA0040 inhibited apoptosis and promoted cell cycle progression, while knockdown of KIAA0040 showed the opposite results. Representative histograms are presented below.

Statistical significance was tested using one-way ANOVA (Dunnett’s tests) for multiple comparison and two-tailed t-tests. ***P* < 0.01 and ****P* < 0.001.


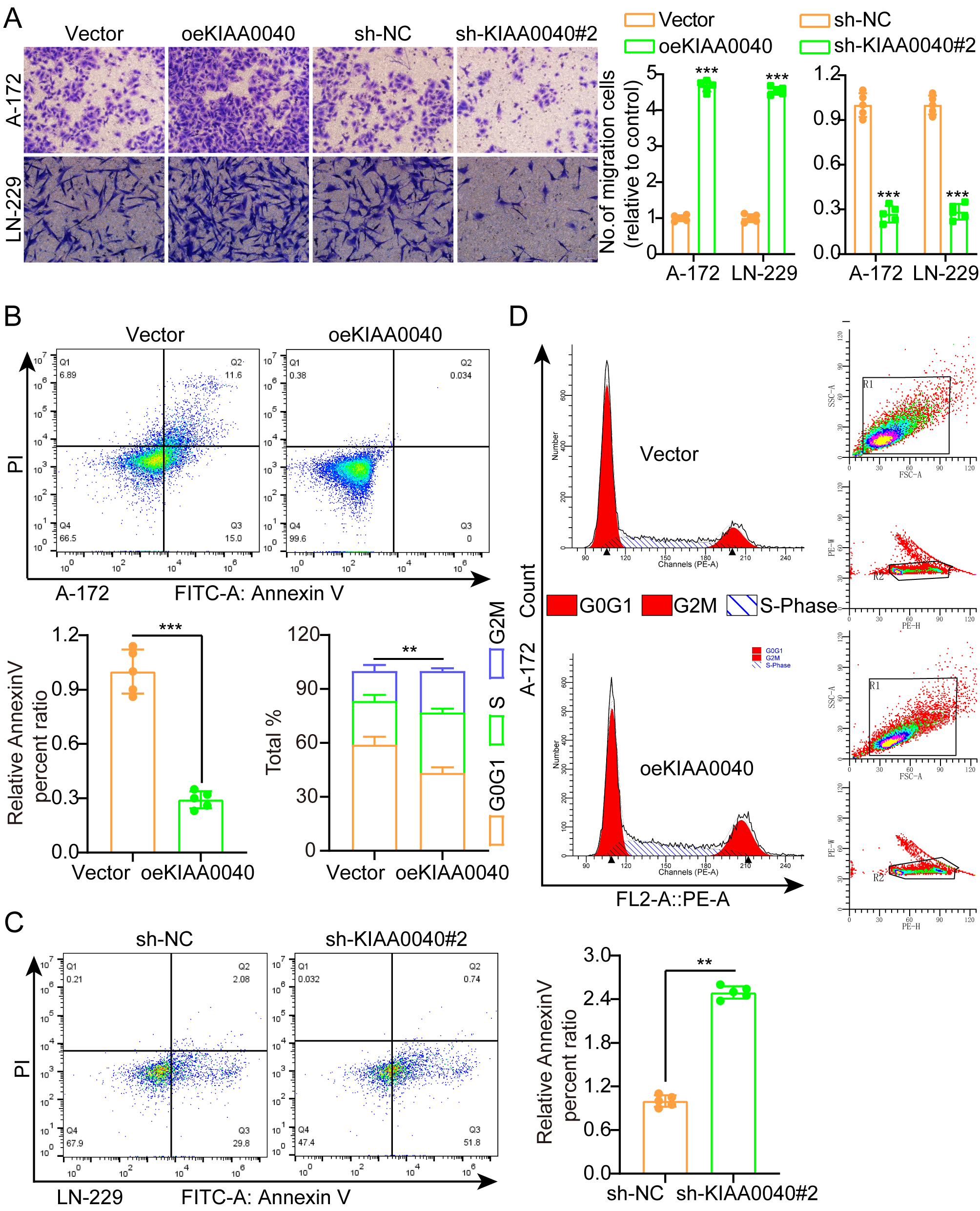


Supplementary Figure 3: KIAA0040 inhibits apoptosis and promotes cell cycle through JAK2-STAT3 signaling pathway.

**A.** Flow cytometry results showed that knockdown of KIAA0040 inhibited cell cycle progression. Representative histograms are presented below.

**B-C.** Flow cytometry results showed that overexpression of KIAA0040 inhibited apoptosis and promoted cell cycle progression, while the above phenomenon can be restored by the JAK2-STAT3 signaling pathway inhibitor Bosutinib. Representative histograms are presented below.

Statistical significance was tested using one-way ANOVA (Dunnett’s tests) for multiple comparison and two-tailed t-tests. **P* < 0.05 and ***P* < 0.01.


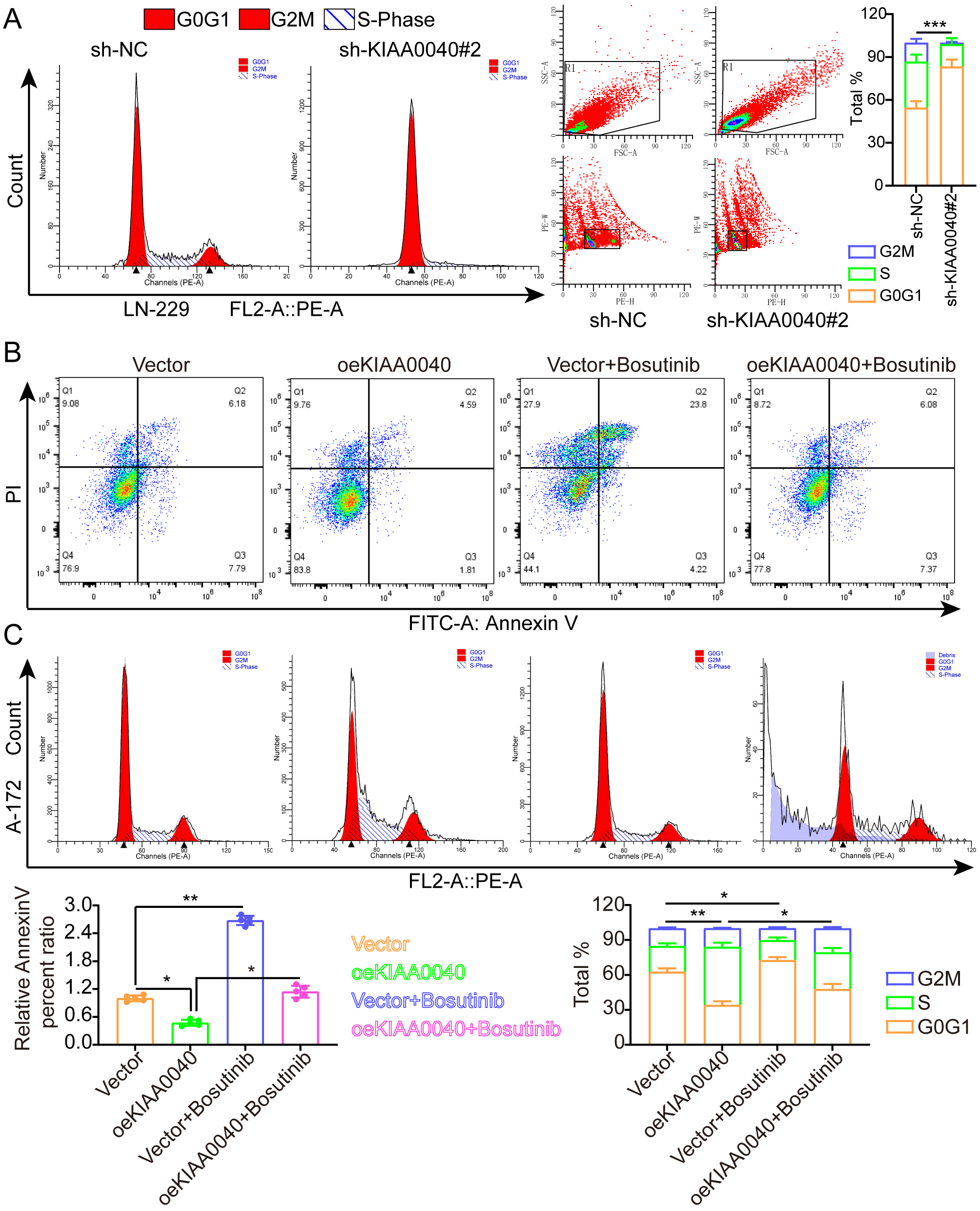


Supplementary Figure 4: Pan-cancer analysis of KIAA0040.

**A.** Flow cytometry results showed that overexpression of KIAA0040 promoted cell cycle progression, while the above phenomenon can be restored by the JAK2-STAT3 signaling pathway inhibitor Bosutinib.

**B.** By analyzing the TCGA database, the results show that KIAA0040 is highly expressed in most tumor tissues.


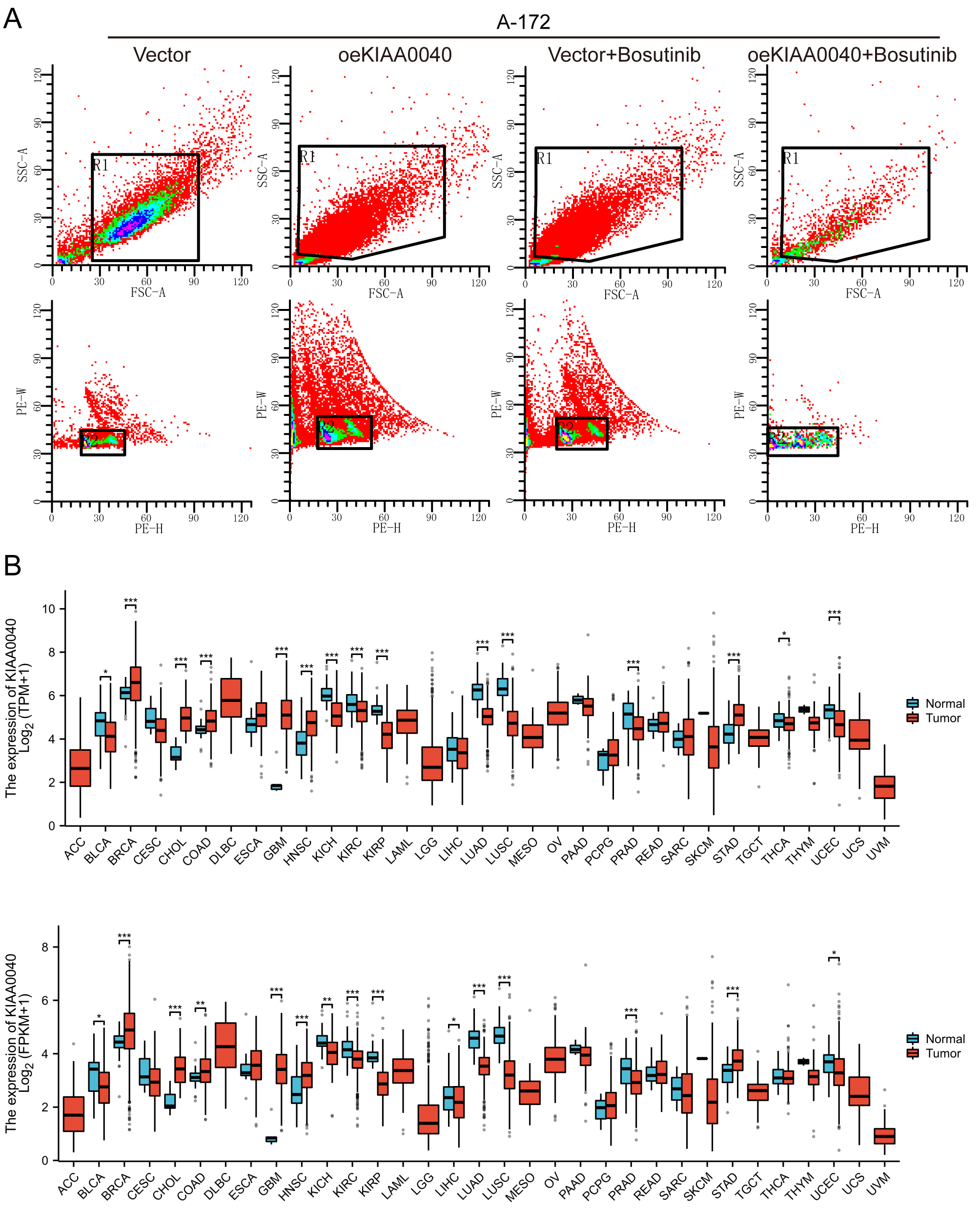


Supplementary Figure 5: Bioinformatics analysis of KIAA0040.

**A.** Left 1: Expression abundance of KIAA0040 in 37 normal tissues in the GTEx database; Right 1: The expression abundance of KIAA0040 in 18 immune cells and PBMC in the HPA database; Right 2: The expression difference of KIAA0040 in solid tumor tissues and para-cancerous tissues in the TCGA database; Right 3: Prognostic value (OS) of KIAA0040 in solid tumors in the TCGA database.

**B.** Expression abundance of KIAA0040 in various cell subpopulations in a low-grade glioma single-cell cohort.


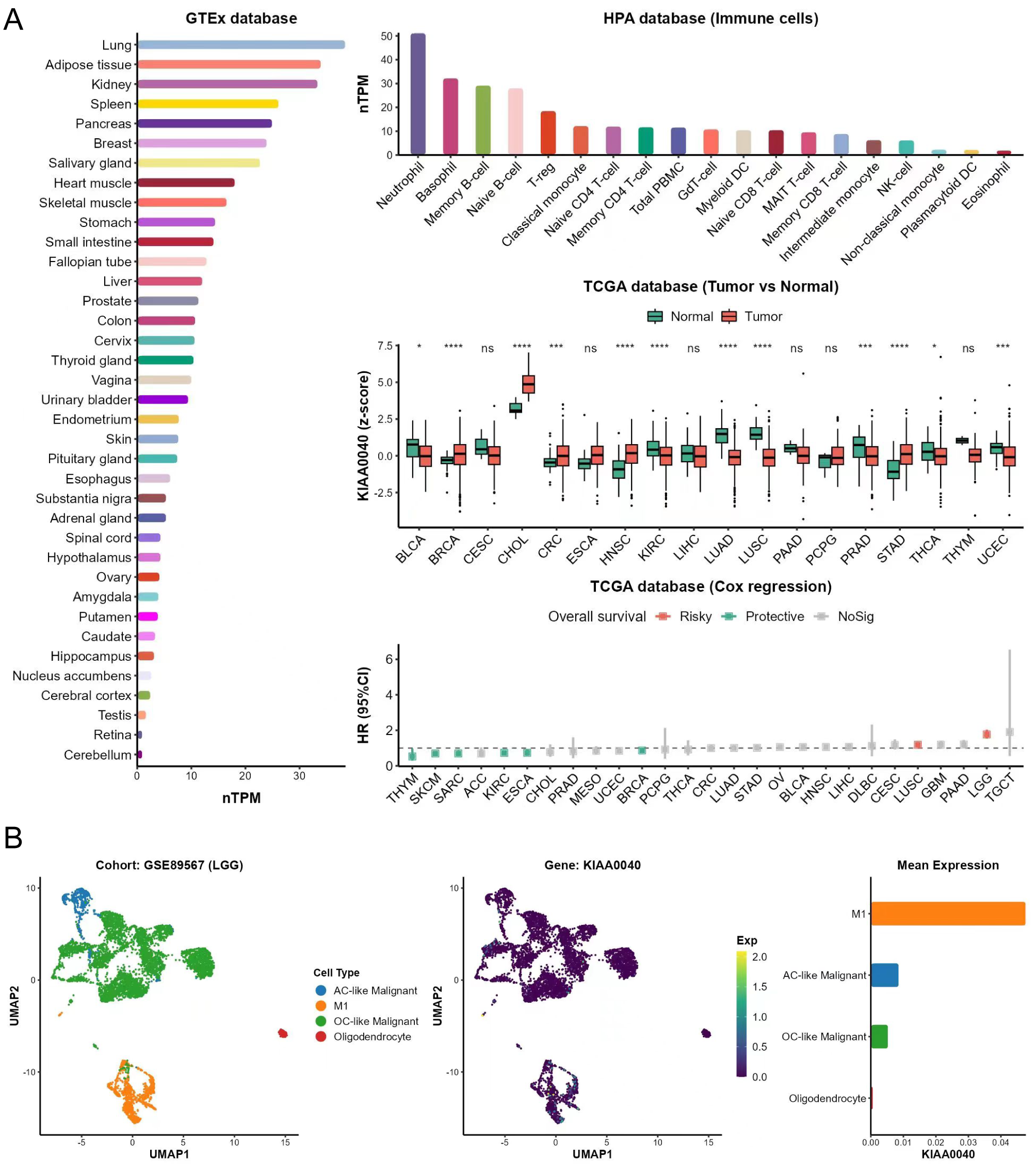


Supplementary Figure 6: Bioinformatics analysis of KIAA0040.

**A.** Expression abundance of KIAA0040 in various cell subpopulations in a high-grade glioma single-cell cohort.

**B.** On the left, in TCGA-LGG, the correlation between KIAA0040 and immune cell infiltration abundance, Fibroblast is calculated by TMPcounter, and other immune cells are deconvolved by the CIBERSORT algorithm. Red represents a significant positive correlation, green represents a significant negative correlation, and gray represents no correlation. On the right, in TCGA-GBM, the correlation between KIAA0040 and immune cell infiltration abundance, Fibroblast is calculated by TMPcounter, and other immune cells are deconvolved by the CIBERSORT algorithm. Red represents a significant positive correlation, green represents a significant negative correlation, and gray represents no correlation.

**C.** WB was used to detect the expression levels of KIAA0040 in normal brain tissue (NBT) and gliomas of different grades (LGG (G1 + G2): low-grade gliomas; HGG (G3 + G4): high-grade gliomas).

**D.** Expression levels of JAK1 and p-JAK1 were detected by WB between Vector and oeKIAA0040.


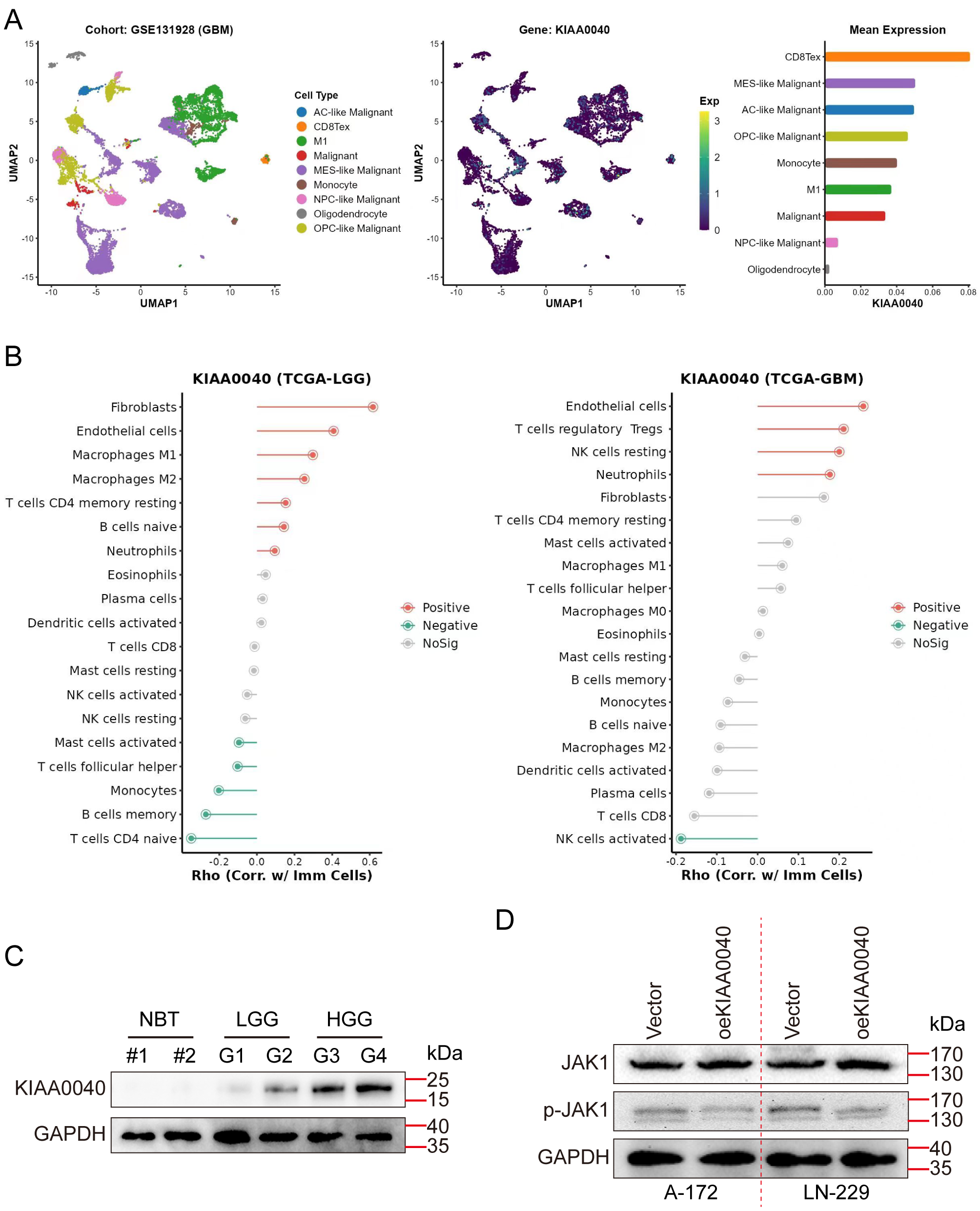


Supplementary Figure 7: Bioinformatics analysis of KIAA0040.

**A.** On the left side, in TCGA-LGG, the correlation analysis between KIAA0040 and all protein-coding genes is first done, and the genes are sorted in descending order based on the correlation coefficient. Finally, the GSEA enrichment analysis of 50 Hallmark pathways is done. Red represents a significant positive correlation, green represents a significant negative correlation, and gray represents no correlation. On the right side, in TCGA-GBM, the correlation analysis between KIAA0040 and all protein-coding genes is first done, and the genes are sorted in descending order based on the correlation coefficient. Finally, the GSEA enrichment analysis of 50 Hallmark pathways is done. Red represents a significant positive correlation, green represents a significant negative correlation, and gray represents no correlation.


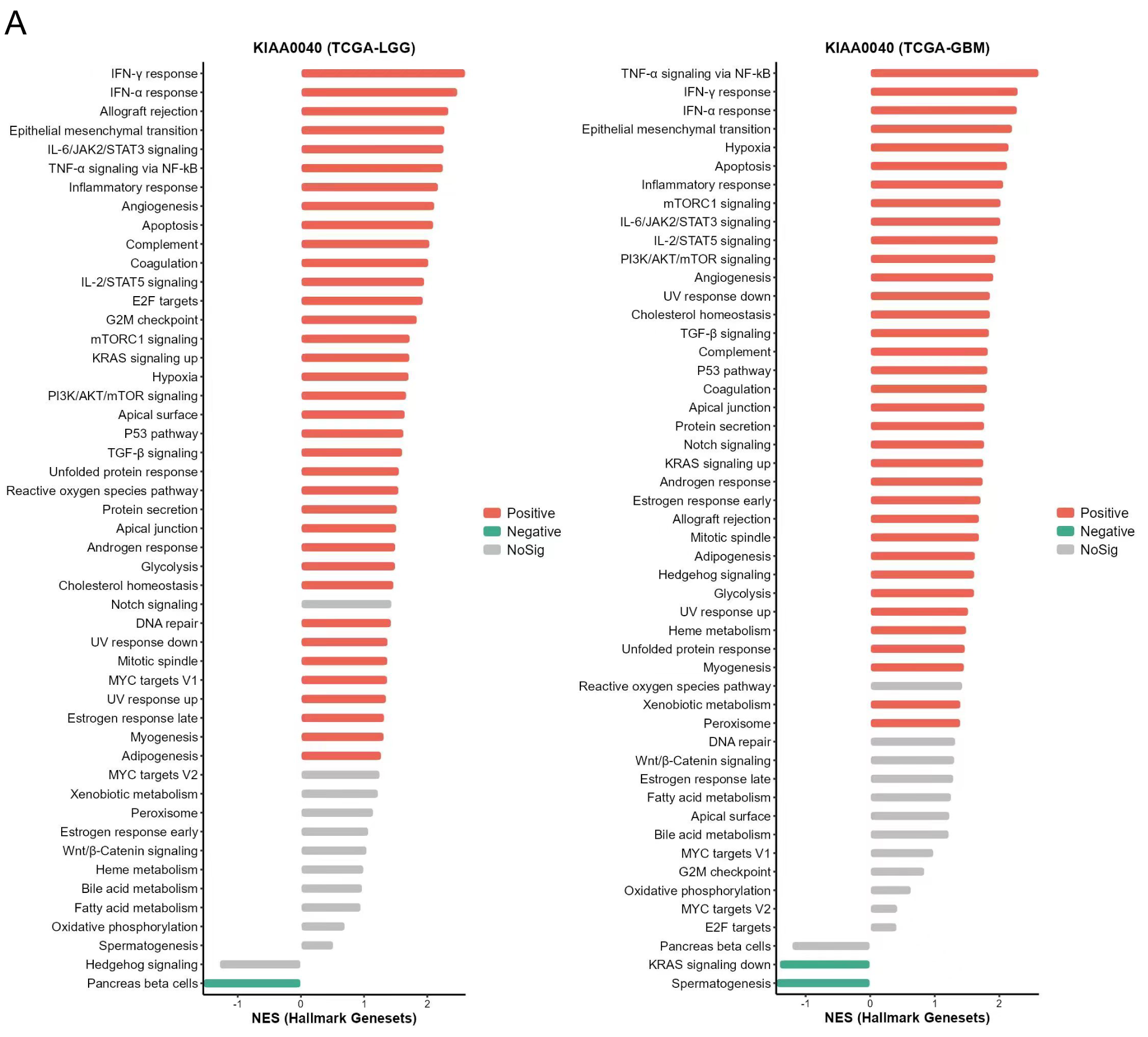

Supplement: Supplementary file 1 — Figure S1. [file JCMM-28-e18332-s001.docx]
